# METTL1 in human cancers: recognition of their functions, mechanisms and therapeutic value

**DOI:** 10.3389/or.2025.1637372

**Published:** 2025-07-30

**Authors:** Xinyu Zhang, Yuan Chen, Min Li, Xiaomeng Zhou, Qingcui Song

**Affiliations:** ^1^ School of Clinical Medicine, Shandong Second Medical University, Weifang, Shandong, China; ^2^ Department of Precision Biomedical Key Laboratory, Liaocheng People’s Hospital, Liaocheng, Shandong, China; ^3^ Shandong Provincial Key Medical and Health Laboratory of Precision Medicine for Aging Intervention and Active Health, Liaocheng, China; ^4^ Department of Oncology, Liaocheng People’s Hospital, Liaocheng, Shandong, China

**Keywords:** METTL1, cancer, RNA methylation, therapeutic target, tumor mechanism

## Abstract

Methyltransferase-like 1 (METTL1) is a methyltransferase that modulates the RNA methylation process and has been increasingly investigated in cancer research over the past decade. The review aims to summarize the diverse roles of METTL1 in various cancers, focusing on the mechanisms underlying tumorigenesis, progression, and metastasis. Furthermore, the therapeutic value and targeting strategies for METTL1 are also discussed to provide the foundation for further development of METTL1-targeted therapies. The article integrates recent research findings to highlight significant discoveries regarding METTL1, emphasizing its potential as a therapeutic target in cancer treatment.

## 1 Introduction

Methyltransferase-like 1 (METTL1) is a crucial enzyme that regulates RNA modifications after transcription, particularly the methylation of guanosine at the N^7^ position (m^7^G) (1–3). This modification serves as a key factor in the molecular networks that modulate RNA metabolism in terms of stability and translation, thereby influencing cellular function (4–6). Furthermore, METTL1, facilitated by its cofactor WD repeat domain 4 (WDR4), primarily modifies transfer RNA (tRNA) by adding methyl groups, which are crucial for tRNA function and subsequent protein synthesis (7–9). In cancer contexts, the METTL1-WDR4 complex is frequently overexpressed and drives malignant progression and therapy resistance by regulating tRNA m^7^G modifications (10). Moreover, the m^7^G modification enhances tRNA stability and promotes tRNA’s interaction with ribosomes. This, in turn, alters subsequent protein synthesis and cellular homeostasis (11, 12). Additionally, studies have shown that METTL1 can modifies messenger RNAs (mRNAs), increasing their stability and translation efficiency. Notably, it stabilizes oncogene mRNAs, such as Cyclin-Dependent Kinase 14 (CDK14), which accelerates cancer cell proliferation (13, 14). The diverse functions of METTL1 highlight its importance in maintaining normal cellular processes and underscore its potential as a valuable therapeutic target for cancers associated with disrupted RNA methylation.

In addition to its role in RNA modification, METTL1 is also implicated in cancer development (15–17). Elevated levels of METTL1 correlates with poor prognosis of various cancers, including bladder cancer (BLCA) (18, 19), hepatocellular carcinoma (HCC) (15, 20), lung cancer (8), colon cancer (21), liver cancer (22), and glioma (23). Evidence indicates that overexpression of METTL1 promotes the proliferation, migration, and invasion of cancer cells, suggesting that it contributes to cancer development (15, 19, 24). Moreover, METTL1 is positively associated with immune cell infiltration in tumor microenvironments (TME). Therefore, it likely modulates tumor biology and immune response, making it a potential target for therapeutic intervention in cancer (25).

The aim of this review is to dissect the multifaceted roles of METTL1 in cancer biology, focusing on its mechanisms of action and therapeutic potential. Several studies have demonstrated that METTL1 may serve as a robust biomarker of cancer prognosis and function as a potential therapeutic target (17, 22, 26). Therefore, it is imperative to review the current understanding of its functions in different cancers. This review specifically examines the effect of METTL1 on tumorigenesis by modulating RNA metabolism and signaling pathways. The findings presented here highlight potential therapeutic targets related to METTL1 for cancer treatment.

### 1.1 Molecular structure of METTL1

Research has shown that METTL1, belonging to the methyltransferase-like protein family, participates in the RNA modification by catalyzing the addition of a methyl group to the N^7^ position of guanosine, which is essential to the formation of N^7^-methylguanosine (m^7^G) (20, 27, 28). This modification influences the stability and function of various RNAs, particularly tRNAs (20, 29) and mRNAs (30–32). Moreover, METTL1 interacts with WDR4, altering its structural conformation and leading to the formation of a heterodimeric complex that is critical for its methyltransferase activity (1, 7). Evidence from emerging studies has uncovered the crystal structure of the METTL1-WDR4 complex, demonstrating that WDR4 functions as a scaffold that stabilizes the interaction between METTL1 and its tRNA substrates (9, 12, 33). METTL1-WDR4 complex modifies tRNA m^7^G46 through unique structures and dynamic mechanisms. It has a sailboat shape, with METTL1’s Rossmann-fold core and WDR4’s β-propeller structure facilitating tRNA binding. WDR4 anchors the tRNA T-arm, while conformational rearrangements in METTL1 enable recognition of the tRNA variable loop, bending the tRNA and positioning the G46 base into the catalytic pocket. The complex specifically targets the tRNA elbow region through shape complementarity (9). From a mechanistic perspective, the N-terminus of METTL1 coordinates the binding of cofactors S-adenosylmethionine/S-adenosylhomocysteine (SAM/SAH), induces conformational changes in tRNA, and activates the catalytic loop (D163/D199/E240) to facilitate proton transfer and methylation, during which the initially disordered N-terminal region adopts an ordered structure upon substrate binding; notably, phosphorylation at S27, mediated by AKT kinase, serves as a regulatory switch that blocks SAM binding and consequently suppresses methyltransferase activity (12). In human liposarcoma models, overexpression of the phosphorylation-mimetic mutant (METTL1-S27D) significantly compromised methyltransferase function yet effectively cooperated with AKT to drive sarcomagenesis, and the catalytically dead mutant (L160A/D163A) similarly retained oncogenic potential, confirming that METTL1-mediated tumor promotion operates independently of its methyltransferase activity. Further research demonstrates that METTL1 binds the multi-tRNA synthetase complex (MSC) to enhance tRNA aminoacylation efficiency, alleviating translation limitation induced by AKT activation and thereby supporting protein synthesis and tumor growth, revealing the molecular basis for METTL1’s non-canonical, methyltransferase-independent role in promoting sarcomagenesis (34).

**FIGURE 1:**
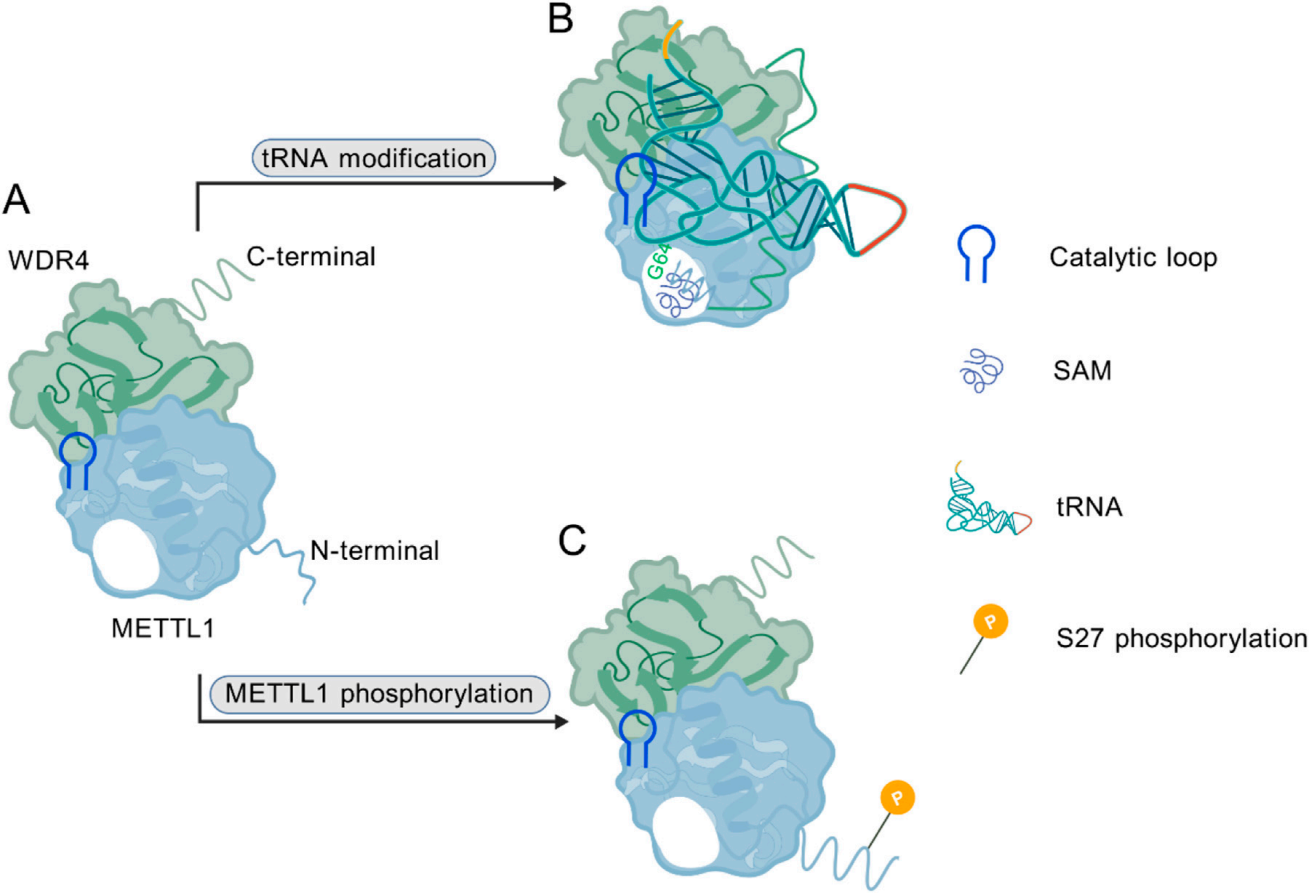
**(A)** METTL1-WDR4 heterodimer complex. **(B)** The METTL1 N-terminus orchestrates cofactor SAM binding, induces tRNA conformational changes, and activates the catalytic loop to facilitate proton transfer and methylation. **(C)** Phosphorylation at the S27 site blocks SAM binding and acts as a regulatory switch. (Created with BioGDP.com).

These structural insights reveal the conformational dynamics underlying the catalytic mechanism of the METTL1-WDR4 complex, and they provide a molecular basis for its involvement in cancer pathogenesis (Figure 1). Understanding the structural details of the METTL1-WDR4 interactions may uncover key molecular mechanisms by which METTL1 contributes to the pathogenesis of various diseases, including cancer (32, 35).

### 1.2 The relationship between METTL1 and cancer

#### 1.2.1 Expression patterns of METTL1 in different types of cancer

**FIGURE 2:**
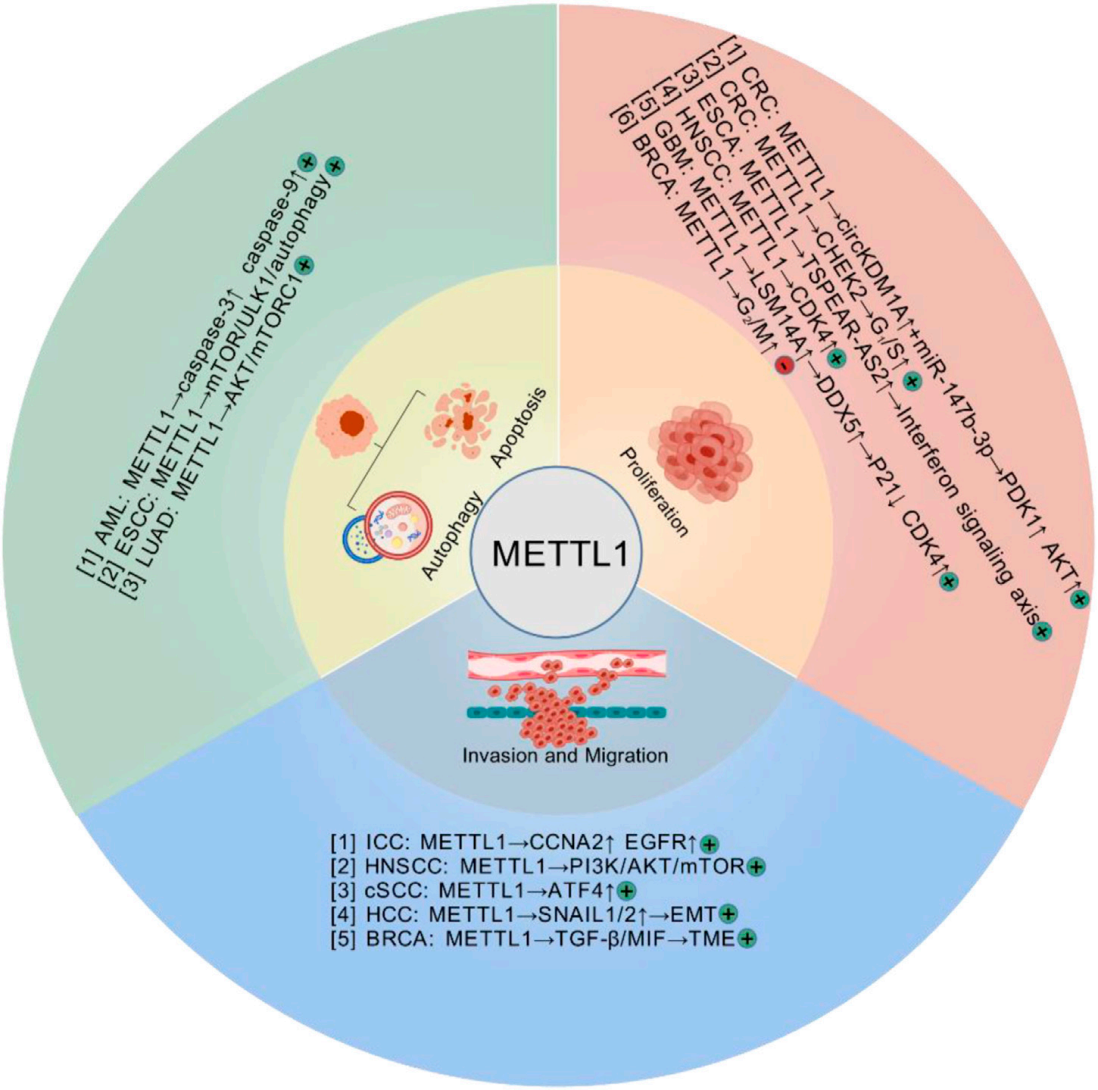
The biological functions and related mechanisms of METTL1 in cancer, including tumor cell proliferation, migration and invasion, apoptosis and autophagy. (+: Promote tumor progression; −: Inhibit tumor progression) (Created with BioGDP.com).

METTL1, a methyltransferase involved in the m^7^G modification of RNA, is differentially expressed across various cancer types (Table 1). Previously, METTL1 was found to be overexpressed in diverse malignancies, including colorectal cancer (CRC) (36, 37), HCC (15, 20, 38), and esophageal cancer (ESCA) (26). Its high expression has been correlated with poor prognosis. In HCC, METTL1 expression is closely associated with tumor malignancy and poor prognosis. Clinical data analysis revealed that high METTL1 expression correlates significantly with larger tumor size, elevated serum AFP levels, tumor vascular invasion, and reduced survival rates. METTL1 was confirmed as an independent prognostic factor for unfavorable outcomes in two independent cohorts (15). Furthermore, in the context of HCC, WDR4 plays a crucial role in functionally interacting with METTL1, thereby enhancing METTL1 expression stability. This interaction is pivotal for the regulation of m^7^G tRNA methylation on target transcripts, which in turn promotes tumor advancement (39). In lung cancer, METTL1/WDR4-mediated m^7^G tRNA modification functions as an oncogenic driver through changing translational efficiency of m^7^G tRNA codon-enriched mRNAs (8). METTL1 is aberrantly elevated in acute myeloid leukemia (AML) and drives leukemogenesis by enhancing tRNA m^7^G modification, stabilizing tRNA to promote translation efficiency and cell proliferation, while its depletion disrupts these processes and induces apoptosis, highlighting its potential as a therapeutic target in AML (40). Single nucleotide polymorphisms (SNPs) within the METTL1 gene could serve as potential biomarkers for the identification of at-risk populations for neuroblastoma. This is supported by evidence suggesting a correlation between variations in genes associated with m^7^G modifications, including METTL1 SNPs, and the risk of developing neuroblastoma (41, 42). While METTL1 predominantly acts as an oncogenic driver in most malignancies, emerging evidence reveals its context-dependent tumor-suppressive functions in specific cancer types. Recent research has indicated that higher METTL1 expression is associated with improved patient survival compared to lower expression in gastric cancer patients (43). Hypoxia-induced HIF-1α downregulates the transcription of METTL1 in CRC cells. Such downregulation leads to a notable reduction in m^7^G modifications, thereby expediting the progression of CRC (37). The highly heterogenous expression profile of METTL1 in various cancers suggests that it may serve as a biomarker of cancer progression and a target for therapeutic intervention. We have summarized the latest findings on the function and related mechanisms of METTL1 in diseases (Figure 2).

**TABLE 1:** Expression of METTL1 in cancer patients.

Tumor type	METTL1 expression level	Model	Role	Phenotype
BLCA (18, 19)	Highly expressed	BCa tissues and cell lines	Promote	Proliferation and metastasis
HCC (15, 20, 38)	Highly expressed	HCC tissues and cell lines	Promote	Proliferation and metastasis
Lung cancer (8)	Highly expressed	Lung cancer tissues	Promote	Proliferation and metastasis
Colon cancer (21)	Lowly expressed	Cisplatin-resistantCC cells	Promote	Cytotoxic effects
Gastric cancer (43)	Highly expressed	Gastric cancer tissues	Promote	Immune evasion
Glioma (23)	Highly expressed	Glioma tissues	Promote	Proliferation
ESCA (26)	Highly expressed	ESCC tissues	Promote	Tumorigenesis
CRC (36, 37)	Highly expressed	CRC cells	Promote	Proliferation and metastasis
HNSCC (35)	Highly expressed	HNSCC tissues and cell lines	Promote	Proliferation and metastasis
Glioblastoma (48)	Highly expressed	GBM cells	Promote	Proliferation and metastasis
AML (40)	Highly expressed	AML clinical samples	Promote	Leukaemogenesis
ICC (29)	Highly expressed	ICC cells	Promote	Survival and progression
cSCC (59)	Highly expressed	cSCC tumors and cells	Promote	Survival, migration, invasion
ccRCC (62)	Highly expressed	ccRCC	Promote	Progression
Prostate cancer (63)	Highly expressed	Prostate cancer tissues and cell lines	Promote	Tumorigenesis
Ameloblastoma (57)	Highly expressed	AM tissues and cell lines	Promote	Growth
OSCC (47)	Highly expressed	OSCC tissues and cell lines	Promote	Proliferation

#### 1.2.2 The relationship between METTL1 and cancer cell proliferation

In recent years, there has been renewed interest in studying the role of METTL1 in cancer growth and progression (15, 35, 44). Notably, METTL1 facilitates cell proliferation by modulating the stability and translation of mRNAs via the m^7^G modification (13, 14). For instance, METTL1 significantly enhances the stability of circKDM1A by recognizing its GG motif and catalyzing m^7^G modification. This modification reduces the minimum free energy (MFE) of the RNA secondary structure and prolongs its half-life. The stabilized circKDM1A acts as a miRNA sponge in the cytoplasm by specifically adsorbing miR-147b-3p through binding to the Argonaute RISC Catalytic Component 2 (AGO2) protein. This interaction alleviates the inhibitory effect of miR-147b-3p on its target gene, Pyruvate Dehydrogenase Kinase 1 (PDK1). The upregulated PDK1 further phosphorylates and activates AKT Serine/Threonine Kinase (AKT)signaling pathway, driving CRC progression (36). Emerging studies show that METTL1 promotes CRC cell proliferation and G_1_/S phase transition through via a CHEK2-dependent mechanism (45). Similar observations have been made in ESCA, where METTL1 enhanced the proliferation and migration by interacting with TSPEAR-AS2, a lncRNA that activates the interferon signaling pathway (46). Another investigation showed that METTL1 facilitated the growth of head and neck squamous cell carcinoma (HNSCC) cells by stabilizing Cyclin Dependent Kinase 4 (CDK4) mRNA, a critical regulator of the cell cycle (35). Recent studies in oral squamous cell carcinoma (OSCC) demonstrate that METTL1 overexpression correlates with poor prognosis. It promotes tumor proliferation by catalyzing m^7^G modification on NEK1 mRNA, which enhances its stability and ultimately induces G_1_/S phase transition (47). METTL1 enhances LSM14A mRNA Processing Body Assembly Factor (LSM14A) mRNA stability and translation by m^7^G methylating it in a complex with WDR4, increasing LSM14A protein expression. In the G_1_/S phase, LSM14A interacts with RNA helicase DDX5 in the cytoplasm, inhibiting its degradation and stabilizing its levels. This accumulation of DDX5 promotes G_1_/S transition by downregulating P21 and upregulating CDK4, while activating migration-related proteins like Matrix Metallopeptidase2/9 (MMP2/9), driving glioblastoma cell proliferation (48). Similarly, METTL1 promotes the progression of AML by regulating tRNA m^7^G modification through two synergistic mechanisms: (1) tRNA stability regulation: METTL1 knockout significantly reduces m^7^G modification at position 46 of tRNAs14. This reduction makes tRNAs more susceptible to degradation by RNase A/T1. Consequently, tRNA abundance decreases, and abnormal accumulation of tRNA-derived small RNA fragments occurs; (2) protein translation control: Loss of m^7^G modification suppresses global translation efficiency by reducing ribosome loading. This leads to decreased synthesis of pro-survival proteins, which causes G_1_-phase cell cycle arrest and increased apoptosis in AML cells (40). Although METTL1 has been widely characterized as an oncogenic driver across multiple malignancies, recent studies challenge this unidimensional perspective. Paradoxically, METTL1 demonstrates context-dependent tumor-suppressive activity, particularly in cancers with specific molecular vulnerabilities—such as BRCA1-deficient breast cancer (BRCA) or IDH-mutant gliomas—where it restricts tumor progression through mechanisms involving tRNA modification-mediated cell cycle arrest (G_2_/M phase prolongation) and enhanced genome stability maintenance (49). The data reviewed here reveal that METTL1 exerts context-dependent roles in cancer biology, either promoting or suppressing tumorigenesis through m^7^G-mediated regulation of RNA metabolism, which dynamically impacts cancer cell proliferation, survival, and progression.

#### 1.2.3 The role of METTL1 in apoptosis and autophagy

In recent years, METTL1 has revealed its unique and complex role in regulating apoptosis and autophagy. It exerts a core function by influencing m^7^G modification of specific RNAs, thereby regulating cellular survival and death decisions. Regarding apoptosis, METTL1 promotes the expression of caspase-3 and caspase-9 to activate apoptotic signaling pathways (40). It also induces mitochondrial membrane potential depolarization by regulating membrane potential stability (50), and affects the expression of antioxidant genes to modulate reactive oxygen species (ROS)-mediated oxidative stress and apoptosis activation (51). This functional complexity parallels other regulatory proteins like Trim45—an E3 ubiquitin ligase that precisely controls protein degradation through the ubiquitin-proteasome system—which similarly impacts cell fate decisions in cancers including cervical cancer and glioblastoma (52). In terms of autophagy, METTL1 impacts tRNA translation efficiency via m^7^G modification, thereby regulating the activity of the ULK1 complex and the mTOR signaling pathway. For instance, in esophageal squamous cell carcinoma, METTL1 suppresses the translation of oncogenic transcripts and promotes autophagy-associated cell death (26, 53), while in lung adenocarcinoma, it inhibits autophagy through the AKT/mTORC1 pathway (54). The expression level of METTL1 exhibits a threshold effect on cell fate; high expression influences tumor prognosis by promoting apoptosis or inhibiting autophagy (22). By regulating downstream pathways like PI3K-Akt, METTL1 acts as a molecular switch determining cell fate choice. A comprehensive analysis of current research demonstrates that METTL1 influences cell fate through multiple mechanisms, including epigenetic modifications, signaling pathway regulation, and metabolic reprogramming, revealing its complex role in disease contexts.

#### 1.2.4 The impact of METTL1 on tumor cell migration and invasion

The involvement of METTL1 in tumor metastasis is increasingly being recognized (20, 29, 55). In various cancer types, METTL1 enhanced the proliferation and invasion of tumor cells by activating the translation of mRNAs containing codons decoded by m^7^G-modified tRNAs (28, 29, 56, 57, 58). METTL1-mediated m^7^G tRNA modification selectively promotes oncogenic mRNA translation via codon-frequency-dependent mechanisms to drive intrahepatic cholangiocarcinoma (ICC) progression. This regulatory process operates through two sequential mechanisms: (1) METTL1 deficiency decreases m^7^G-modified tRNA (e.g., LysCTT) abundance, inducing ribosome stalling at high-frequency codons (e.g., AAG) and preferentially suppressing translation of codon-enriched oncogenic transcripts like Cyclin-A2 (CCNA2) and Epidermal Growth Factor Receptor (EGFR); (2) Resultant translational repression reduces protein expression of cell cycle regulators (CCNA2, CDK6) and EGFR signaling components (EGFR, AKT, mTOR), ultimately inhibiting ICC proliferation and invasion (29). In HNSCC, METTL1/WDR4 catalyzes m^7^G modifications on tRNAs (primarily at the “RRGGYYS” motif within the V-loop) to stabilize specific tRNAs, thereby enhancing the efficient translation of codons dependent on m^7^G-modified tRNAs (e.g., ValACC) by ribosomes. When METTL1 is functionally impaired, reduced m^7^G modification levels lead to ribosome pausing at these codon sites, significantly compromising decoding efficiency. This translational impairment is particularly enriched in mRNAs of PI3K/AKT/mTOR pathway-related genes, as their open reading frames exhibit high dependency on m^7^G tRNA-specific codons. METTL1 deficiency diminishes the synthesis of key proteins in this pathway, ultimately suppressing HNSCC cell proliferation and metastasis (56). In cutaneous squamous cell carcinoma (CSCC), METTL1 stabilizes Activating Transcription Factor 4 (ATF4) mRNA and increases its expression via m^7^G methylation. Importantly, restoring ATF4 levels leads to glycolytic metabolic reprogramming in tumor cells and counteracts the anti-tumor effects caused by METTL1 knockdown (59). In HCC with insufficient radiofrequency ablation (iRFA), METTL1 enhances translation of Snail Family Transcriptional Repressor 1(SNAIL1) and Snail Family Transcriptional Repressor 2(SNAIL2), key epithelial-mesenchymal transition (EMT) regulator, thereby driving heat stress-induced metastatic progression (60). M^7^G drives BRCA metastasis via EMT and immunosuppression. High m^7^G synergizes with Transforming Growth Factor Beta 1/Macrophage Migration Inhibitory Factor (TGF-β/MIF) to promote invasion, while METTL1/WDR4 regulate metastasis genes and correlate with advanced stages. The m^7^G-TME classifier identifies aggressive (m^7^G-high + TME-low) and favorable prognosis subgroups (m^7^G-low + TME-high), highlighting m^7^G-TME interplay as a metastasis regulator (61). The associations among METTL1, m^7^G modification, and the metastatic potential of tumors underscore the importance of this enzyme in the broader context of cancer biology.

### 1.3 The role of METTL1 in the TME

The TME is a complex network composed of diverse cell types, extracellular matrix components, and signaling molecules. Interactions among these components influence tumor progression and response to therapy (64–66).

#### 1.3.1 The impact of METTL1 on tumor immunity

Several investigations have indicated that METTL1 plays a role in the TME, influencing tumor immunity (56, 67). In HCC, it stimulates TGF-β2 translation, which subsequently induces the accumulation of polymorphonuclear myeloid-derived suppressor cells (PMN-MDSCs) and inhibits CD8^+^ T cell infiltration, thereby fostering the creation of an immunosuppressive microenvironment (68). In clear cell renal cell carcinoma (ccRCC), the upregulation of METTL1 is associated with disease advancement and a heightened presence of immunosuppressive regulatory T cells (Tregs). The underlying mechanism suggests that METTL1 overexpression may promote tumor immune evasion by establishing an immunosuppressive microenvironment predominantly characterized by Tregs (62). In HNSCC, m^7^G modification drives an immunosuppressive tumor microenvironment through multi-dimensional mechanisms. A prognostic model based on m^7^G-related genes showed that high-risk scores were significantly associated with reduced CD8^+^ T cell infiltration and increased M2 macrophages. This effect was mediated by upregulation of Programmed Cell Death Ligand 1 (PD-L1). Single-cell sequencing revealed 1.8–2.5-fold upregulation of glycolysis genes in m^7^G-high cells, promoting Treg differentiation and CD8^+^ T cell inhibition through lactate accumulation. This study first elucidated the LINC00707/miR-30b-5p/LARP1 axis as a central regulatory pathway in m^7^G-mediated immune suppression (69). Additionally, in CRC, the modification of PKM2 mRNA by METTL1 through m^7^G enhances the expression of PKM2, leading to the establishment of a positive feedback loop involving histone H3K9 lactylation (H3K9la), METTL1, and PKM2. This loop functions as follows: PKM2 facilitates glycolysis and lactate production, while lactate subsequently activates METTL1 through H3K9la. This regulatory axis promotes immune evasion in CRC by transcriptionally activating CD155, thereby reinforcing the METTL1-m^7^G-dependent signaling pathway of PKM2 (70). To further support the immunosuppressive role of METTL1, a prior study demonstrated that it regulates several chemokines, such as C-X-C Motif Chemokine Ligand 8 (CXCL8), which modulate the recruitment of immunosuppressive cells (71). Moreover, modifies the immune landscape of tumor cells, altering the interactions between the tumor and stromal cells, hindering anti-tumor immunity (56). METTL1 orchestrates tumor immune evasion across multiple cancers by modulating immunosuppressive microenvironments through distinct mechanisms, positioning it as a promising therapeutic target to enhance immunotherapy efficacy.

#### 1.3.2 Relationship between METTL1 and immune checkpoint molecules

Prior investigations have uncovered an association between METTL1 and immune checkpoint molecules, further supporting its role in tumor immunology (71, 72). Moreover, the expression level of METTL1 can alter the transcription of immune checkpoints such as PD-1 and Cytotoxic T-Lymphocyte-Associated Protein 4(CTLA-4), which modulates T cell responses (16, 73). A study on gastric cancer showed that overexpression of METTL1 promoted CTLA-4 and PD-1 expression, suggesting that METTL1 may facilitate immune evasion by enhancing the expression of these inhibitory checkpoints (43). Other scholars have demonstrated that METTL1 can modify the immune microenvironment by regulating the infiltration of various immune cell types, including T cells and macrophages, thereby affecting anti-tumor immune responses (72, 74). This cross-communication suggests that inhibiting METTL1 may improve the efficacy of immune checkpoint inhibitors and hence, the outcomes of patients receiving immunotherapy. Overall, the available evidence confirms that METTL1 is associated with immune checkpoints, making it an important regulator of cancer immunology and a therapeutic target.

#### 1.3.3 METTL1 and stromal cells in the TME

The TME also contains stromal cells, which include fibroblasts and immune cells (75–77). Recent studies have demonstrated that METTL1 modulates the behavior of stromal cells in the TME (25, 56, 78).

METTL1 regulates gene expression in stromal cells: in CRC, METTL1 modulates the expression of PKM2 through m^7^G mRNA modification, thereby promoting metabolic reprogramming and immune escape in tumor cells (70). Additionally, METTL1 enhances the translation of specific oncogenes via m^7^G tRNA modification, facilitating stromal cell functionality and gene expression within the TME (56). These findings highlight METTL1’s dual role in both tumor cells and stromal compartments.

METTL1 influences stromal cell differentiation and function: METTL1 significantly impacts stromal cell differentiation and specific functional states, such as immunosuppressive activity and cytokine secretion profiles, across multiple cancer types. For example, METTL1 upregulation drives the differentiation of tumor-associated stromal cells, which in turn amplifies their immunosuppressive functions and reduces CD8^+^ T cell infiltration in HCC (68). Consequently, this highlights METTL1’s critical role in facilitating immune evasion by modulating stromal cell behavior. METTL1 orchestrates TME formation and progression by regulating stromal cell gene expression and functional dynamics. These mechanisms position METTL1 as a pivotal therapeutic target for disrupting tumor-stroma crosstalk.

### 1.4 METTL1’s signaling pathways and regulatory network

#### 1.4.1 Mechanisms of METTL1-mediated RNA methylation in cancer

As a pivotal m^7^G methyltransferase, the biological functions of METTL1 primarily manifest in its m^7^G modification of RNA, participating in the m^7^G modification processes of diverse RNA species—including tRNA, mRNA, and ribosomal RNA (rRNA)—and playing a critical role in the progression of multiple malignancies (18, 20, 58, 67).

Catalytic mechanism of METTL1 in tRNA m^7^G modification: AS the core component of the methyltransferase complex, METTL1 collaborates with WDR4 to introduce m^7^G methylation at position 46 of the variable loop (G46) in tRNAs. This modification significantly enhances the structural stability of specific tRNAs and optimizes ribosomal decoding efficiency by modulating their tertiary conformation. Under METTL1 overexpression, m^7^G-modified tRNAs preferentially recognize complementary codons (e.g., GTG-enriched codons in Growth Arrest And DNA Damage Inducible Alpha (GADD45A) mRNA and CGC codons in RB Transcriptional Corepressor 1 (RB1) mRNA), promoting ribosome translocation on target mRNAs in a codon-biased manner. By accelerating the decoding of m^7^G-associated codons, METTL1 selectively enhances the translational efficiency (TE) of GADD45A and RB1—rather than globally regulating all mRNAs. This tRNA modification-mediated translational upregulation ultimately elevates GADD45A and RB1 protein levels, activating cell cycle checkpoints to induce G_2_/M phase arrest, thereby suppressing BRCA progression (49). METTL1 maintains tRNA m^7^G modification to inhibit the biogenesis of 5′TOG fragments, thereby regulating specific translational programs and suppressing interferon pathway activation. Targeting METTL1 can enhance immunotherapy efficacy by reprogramming the TME. This approach provides a novel potential therapeutic strategy for prostate cancer treatment (63).

Role of METTL1 in mRNA methylation and cancer pathogenesis: METTL1-mediated mRNA methylation also plays a key role in tumorigenesis (3, 35). For instance, METTL1 stabilizes Ribosomal RNA Processing 9, U3 Small Nucleolar RNA Binding Protein (RRP9) mRNA via m^7^G modification, thereby promoting tumor cell proliferation and metastasis in CRC.

Structural Insights into METTL1-Dependent miRNA Maturation: Mechanistic studies demonstrate that the METTL1/WDR4 complex recognizes G-rich regions in pri-miR-760 and introduces m^7^G modifications via SAM-dependent catalytic reactions. These modifications enhance the cleavage efficiency of the Drosha/DGCR8 complex, accelerating miR-760 maturation. This m^7^G-dependent regulatory axis culminates in mature miR-760-mediated degradation of the tumor suppressor ATF3 mRNA, driving BLCA cell proliferation, migration, and *in vivo* metastasis (18). The METTL1-mediated m^7^G modification at the G11 site of the let-7e-5p precursor, also known as primary microRNA (pri-miRNA), enhances DROSHA cleavage efficiency by disrupting the precursor’s inhibitory G-quadruplex structure. This methylation suppresses G-quadruplex formation through interference with Hoogsteen base-pairing, as confirmed by 7-deazaguanosine (DAG) substitution restoring processing efficiency. Loss of METTL1 reduces mature let-7e levels by 60%, upregulating the oncogenic target High Mobility Group AT-Hook 2 (HMGA2) and promoting cancer cell migration (79). In short, METTL1 serves as a central regulator of diverse RNA modifications, profoundly influencing tumor initiation and progression. Its multifaceted roles establish METTL1 as a critical target in cancer research, offering promising avenues for therapeutic intervention.

#### 1.4.2 Major METTL1-regulated oncogenic signaling pathways

**FIGURE 3:**
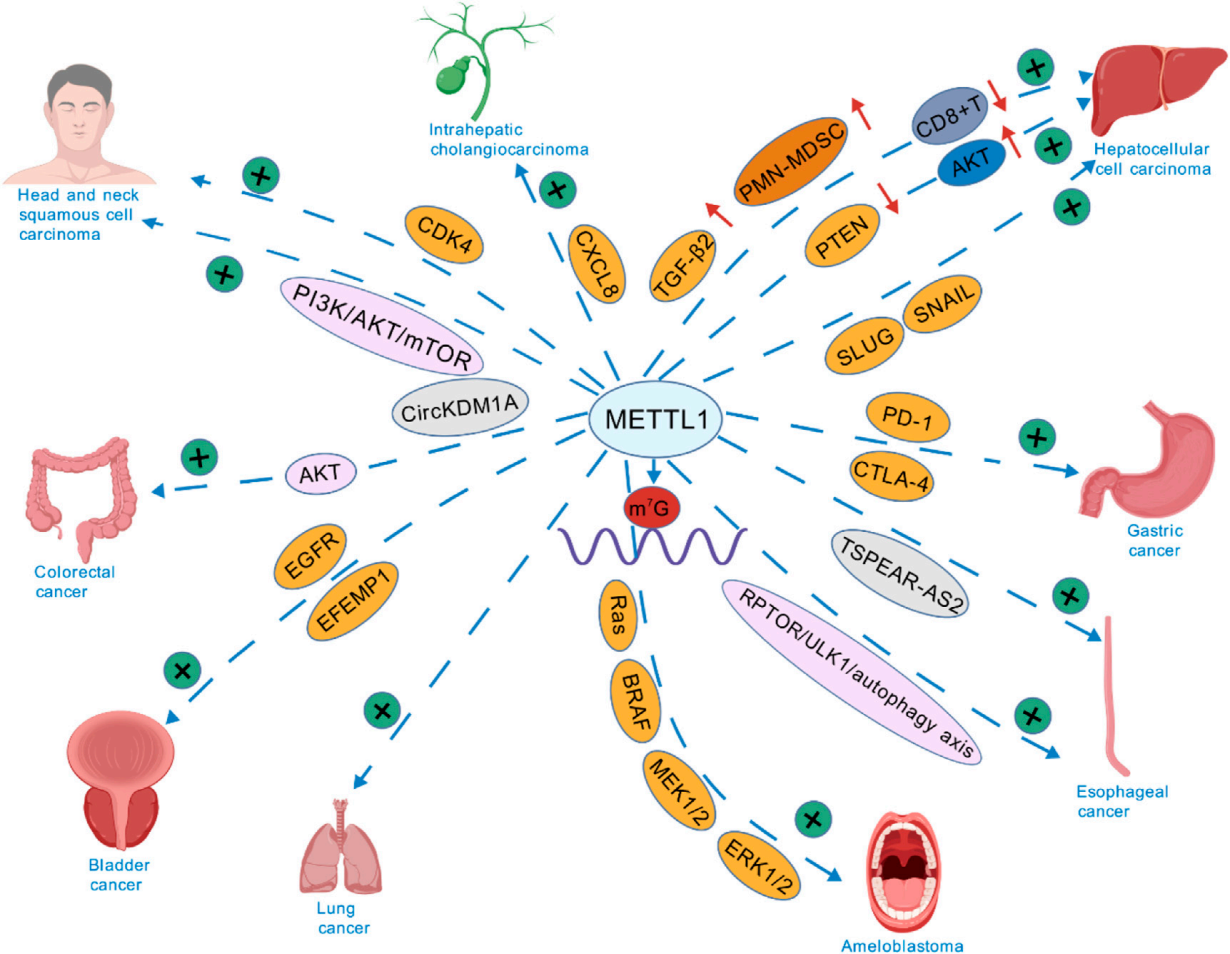
METTL1’s Signaling pathways in various cancers. (Created with BioGDP.com).

The various signaling pathways through which METTL1 regulates cancer progression are shown in Figure 3. The role of PI3K/Akt and MAPK pathways in mediating the effects of METTL1 have been widely documented (15, 57). In HCC, METTL1 overexpression was reported to be correlated with larger tumor sizes and poor prognosis, primarily by activating the PTEN/AKT signaling pathway. Moreover, overexpression of METTL1 resulted in decreased PTEN expression, leading to elevated AKT activity, promoting cell proliferation and migration (15). In HCC, the m^7^G methylation facilitated by METTL1 is responsible for the regulation of circIPP2A2, which functions as a molecular scaffold. This interaction enhances the association between Hornerin and PI3K, subsequently activating the PI3K/AKT/GSK3β signaling cascade. This pathway plays a crucial role in promoting the aggressiveness of HCC (80). Similarly, METTL1 promoted tumor progression by modulating the expression of oncogenic transcripts via the PI3K/AKT/mTOR signaling pathway in HNSCC. METTL1 knockdown induced reduction in m^7^G levels of tRNAs that translate these oncogenic mRNAs, which inhibited tumor growth (56). Additionally, the MAPK pathway has been recognized as a crucial factor in promoting the invasive growth of ameloblastoma. METTL1 enhances translation efficiency of MAPK signaling genes (e.g., Ras, BRAF, MEK1/2, ERK1/2) in ameloblastoma without altering their mRNA levels. RNC-seq profiling shows METTL1 depletion selectively impairs translation of MAPK-related transcripts, while qRT-PCR confirms unchanged transcription. This tRNA-specific modification—distinct from mRNA methylation mechanisms—downregulates translation of downstream effectors (Cyclin D1, MMP2/9, Vimentin), establishing tRNA m^7^G-driven translational control of MAPK pathway activation (57). Collectively, these findings indicate that METTL1 serves as a critical regulator of the PI3K/Akt and MAPK signaling pathways, contributing to the malignant phenotypes of various cancers.

### 1.5 The potential of METTL1 as a target for cancer therapy

#### 1.5.1 Current status of drug development targeting METTL1

METTL1 has emerged as a promising therapeutic target due to its oncogenic role in regulating RNA stability and translation via m^7^G modifications. Current drug development efforts focus on three primary strategies.

Small-molecule inhibitors: A pioneering study first identified METTL1 inhibitors through high-throughput docking and a luminescence-based enzymatic assay, where eleven compounds from three distinct chemotypes demonstrated inhibitory activity in the 40–300 μM range, with adenine derivatives exhibiting high ligand efficiency, highlighting their optimization potential. Molecular dynamics simulations revealed these inhibitors competitively block the binding of the co-substrate SAM to METTL1’s catalytic pocket, with structural validation achieved using a soakable crystal form resolving complexes at 1.85 Å resolution (81). Although these early findings establish a molecular foundation, no compounds have advanced to preclinical animal efficacy studies. However, progress with analogous RNA methyltransferase targets (e.g., METTL3 inhibitors advancing to clinical trials) provides a promising pathway for optimizing METTL1 inhibitors (82, 83).

Nanotherapeutic approaches: The PAE@5-FUts nanocomplex selectively delivers 5-fluorouracil (5-FU) to CRC cells overexpressing METTL1. By silencing METTL1-induced tsRNA-GlyGCC, it restores 5-FU sensitivity (IC_50_ reduction from 28 μM to 6 μM) and suppresses JAK1/STAT6 signaling *in vivo* (84). Although nanotherapies targeting METTL1 demonstrate significant potential in tumor treatment, their clinical translation faces critical challenges: safety requires in-depth evaluation of long-term toxicity risks and impacts on normal tissues (85); delivery efficiency is constrained by tumor microenvironment heterogeneity, necessitating optimization of carrier physicochemical properties (e.g., particle size, surface charge) and production quality (86); tumor-specific targeting demands precise patient stratification and ligand modification (e.g., aptamers or antibodies) to enhance cellular selectivitys (87). Future efforts should prioritize intelligent carrier development (e.g., pH/enzyme-responsive materials) and individualized strategies to address these barriers.

Combination therapies: METTL1 overexpression synergizes with CDK4/6 inhibitors (e.g., abemaciclib) in BRCA by enhancing m^7^G tRNA modification-driven translational activation of GADD45A and RB1, amplifying cell cycle blockade through G_2_/M phase arrest (via Cyclin B1/CDK1 suppression) and partial G_1_/S modulation (via RB1-E2F inhibition). *In vivo* studies showed combined therapy with METTL1 overexpression significantly improved tumor suppression compared to monotherapies, correlating with elevated RB1/GADD45A levels and reduced Ki67. These findings position METTL1-mediated tRNA epitranscriptomic regulation as a novel enhancer of CDK4/6 inhibitor efficacy(49).

#### 1.5.2 Clinical application prospects and challenges

Although METTL1 has several clinical applications as a therapeutic target, there are significant challenges that need to be addressed. For instance, targeting METTL1 may have important therapeutic benefits on various cancers, particularly those characterized by high METTL1 expression, such as BLCA and HCC (15, 19). Researchers have shown that METTL1 can modulate RNA modifications to improve the efficacy of immunotherapies (88–90). Indeed, METTL1 expression altered response to PD-L1 blockade by modulating immune infiltration (16). Its clinical application requires the identification of precise biomarkers that will help to select patient populations who may benefit from METTL1-targeted therapies. Moreover, the complex RNA modification pathways interact with various cellular processes. This interplay highlights the need to fully understand the potential side effects and broader consequences of inhibiting METTL1 activity (91). Furthermore, clinical trial studies are needed to evaluate their safety and efficacy. Overall, the clinical application of METTL1 is yet to be realized and further investigations are needed to resolve the above challenges.

## 2 Conclusion

In recent years, research has documented that METTL1 plays a critical role in cancer biology through multifaceted mechanisms influencing tumorigenesis 13, acting as a vital component of the mRNA methylation machinery that regulates gene expression, RNA stability, and translation efficiency to drive oncogenic processes across various cancer types; this positions METTL1 as a promising therapeutic target, where modulation of its activity could exploit tumor vulnerabilities caused by dysregulation, enabling targeted therapies to inhibit or enhance its function for personalized treatments that improve clinical outcomes and reduce adverse effects 38. However, current research faces significant limitations, particularly the incomplete understanding of specific mechanisms by which METTL1 alters the tumor microenvironment and other oncogenic factors, as these interactions remain poorly elucidated and hinder the development of robust combination therapies 18. Future prospects should thus focus on addressing these gaps through advancing cancer genomics with technologies like CRISPR and RNA sequencing to uncover METTL1’s full role across diverse cancer subtypes, facilitating better patient stratification, while collaborative efforts between basic scientists and clinicians are essential to accelerate discoveries in METTL1-targeted strategies and promote clinical translation, thereby fostering innovations that improve patient outcomes despite existing complexities.

## Glossary

METTL1: Methyltransferase-like 1
**m** ^**7**^ **G**: N^7^-methylguanosine
WDR4: WD repeat domain 4
tRNA: transfer RNA
mRNAs: messenger RNAs
CDK14: Cyclin Dependent Kinase 14
BLCA: bladder cancer
HCC: hepatocellular carcinoma
TME: tumor microenvironments
SAM: S-adenosylmethionine
SAH: S-adenosylhomocysteine
MSC: multi-tRNA synthetase complex
CRC: colorectal cancer
ESCA: esophageal cancer
AML: acute myeloid leukemia
SNPs: single nucleotide polymorphisms
MFE: minimum free energy
PDK1: Pyruvate Dehydrogenase Kinase 1
HNSCC: head and neck squamous cell carcinoma
CDK4: Cyclin Dependent Kinase 4
OSCC: oral squamous cell carcinoma
MMP2/9: Matrix Metallopeptidase2/9
BRCA: breast cancer
ROS: reactive oxygen species
ICC: intrahepatic cholangiocarcinoma
CCNA2: Cyclin-A2
EGFR: Epidermal Growth Factor Receptor
CSCC: cutaneous squamous cell carcinoma
ATF4: Activating Transcription Factor 4
iRFA: insufficient radiofrequency ablation
SNAIL1: Snail Family Transcriptional Repressor 1
SNAIL2: Snail Family Transcriptional Repressor 2
EMT: epithelial-mesenchymal transition
TGF-β: Transforming Growth Factor Beta
MIF: Macrophage Migration Inhibitory Factor
PMN-MDSCs: polymorphonuclear myeloid-derived suppressor cells
ccRCC: clear cell renal cell carcinoma
PD-L1: Programmed Cell Death Ligand 1
CTLA-4: Cytotoxic T-Lymphocyte-Associated Protein 4
rRNA: ribosomal RNA
TE: translational efficiency
RRP9: Ribosomal RNA Processing 9
5-FU: 5-fluorouracil

## References

[1] Cheng W Gao A Lin H Zhang W. Novel roles of METTL1/WDR4 in tumor via m^7^G methylation. Mol Ther - Oncolytics (2022) 26:27–34. 10.1016/j.omto.2022.05.009 35784404 PMC9217986

[2] Wang L Zhou J Kong L Ying G Sha J Yi D Fibroblast-specific knockout of METTL1 attenuates myocardial infarction-induced cardiac fibrosis. Life Sci (2023) 329:121926. 10.1016/j.lfs.2023.121926 37437652

[3] Zhao Y Kong L Pei Z Li F Li C Sun X m7G methyltransferase METTL1 promotes post-ischemic angiogenesis via promoting VEGFA mRNA translation. Front Cell Dev Biol (2021) 9:642080. 10.3389/fcell.2021.642080 34136476 PMC8200671

[4] Ho JJD Man JHS Schatz JH Marsden PA. Translational remodeling by RNA-binding proteins and noncoding RNAs. Wiley Interdisciplinary Reviews RNA (2021) 12(5):e1647. 10.1002/wrna.1647 33694288

[5] Chatterjee B Shen CKJ Majumder P. RNA modifications and RNA metabolism in neurological disease pathogenesis. Int J Mol Sci (2021) 22(21):11870. 10.3390/ijms222111870 34769301 PMC8584444

[6] Du QY Zhu ZM Pei DS. The biological function of IGF2BPs and their role in tumorigenesis. Invest New Drugs (2021) 39(6):1682–93. 10.1007/s10637-021-01148-9 34251559

[7] Weng Q Zhang F Zheng Q. A comprehensive model for tRNA methylation modification studies. MedComm (2020)(2023) 4(6):e402. 10.1002/mco2.402 PMC10590456 37873513

[8] Ma J Han H Huang Y Yang C Zheng S Cai T METTL1/WDR4-mediated m^7^G tRNA modifications and m^7^G codon usage promote mRNA translation and lung cancer progression. Mol Ther (2021) 29(12):3422–35. 10.1016/j.ymthe.2021.08.005 34371184 PMC8636169

[9] Li J Wang L Hahn Q Nowak RP Viennet T Orellana EA Structural basis of regulated m^7^G tRNA modification by METTL1-WDR4. Nature (2023) 613(7943):391–7. 10.1038/s41586-022-05566-4 36599985 PMC11179147

[10] Zhang H Sun F Jiang S Yang F Dong X Liu G METTL protein family: focusing on the occurrence, progression and treatment of cancer. Biomark Res (2024) 12(1):105. 10.1186/s40364-024-00652-3 39289775 PMC11409517

[11] Li R Liu X Deng K Wang X. M7G methylated core genes (METTL1 and WDR4) and associated RNA risk signatures are associated with prognosis and immune escape in HCC. BMC Med Genomics (2023) 16(1):179. 10.1186/s12920-023-01614-8 37528384 PMC10394781

[12] Ruiz-Arroyo VM Raj R Babu K Onolbaatar O Roberts PH Nam Y. Structures and mechanisms of tRNA methylation by METTL1-WDR4. Nature (2023) 613(7943):383–90. 10.1038/s41586-022-05565-5 36599982 PMC9930641

[13] Zhang M Kan D Zhang B Chen X Wang C Chen S P300/SP1 complex mediating elevated METTL1 regulates CDK14 mRNA stability via internal m7G modification in CRPC. J Exp Clin Cancer Res (2023) 42(1):215. 10.1186/s13046-023-02777-z 37599359 PMC10440916

[14] Wang Z Yu P Zou Y Ma J Han H Wei W METTL1/WDR4-mediated tRNA m^7^G modification and mRNA translation control promote oncogenesis and doxorubicin resistance. Oncogene (2023) 42(23):1900–12. 10.1038/s41388-023-02695-6 37185458

[15] Tian QH Zhang MF Zeng JS Luo RG Wen Y Chen J METTL1 overexpression is correlated with poor prognosis and promotes hepatocellular carcinoma via PTEN. J Mol Med (Berl) (2019) 97(11):1535–45. 10.1007/s00109-019-01830-9 31463732

[16] Gao Z Xu J Zhang Z Fan Y Xue H Guo X A comprehensive analysis of METTL1 to immunity and stemness in pan-cancer. Front Immunol (2022) 13:795240. 10.3389/fimmu.2022.795240 35432338 PMC9008260

[17] Zheng P Yang S Ren D Zhang X Bai Q. A pan-cancer analysis of the oncogenic role of methyltransferase-like 1 in human tumors. Neurol India (2024) 72(4):837–45. 10.4103/neurol-india.ni_1354_21 39216043

[18] Xie H Wang M Yu H Wang H Ding L Wang R METTL1 drives tumor progression of bladder cancer via degrading ATF3 mRNA in an m^7^G-modified miR-760-dependent manner. Cell Death Discov (2022) 8(1):458. 10.1038/s41420-022-01236-6 36396627 PMC9672058

[19] Ying X Liu B Yuan Z Huang Y Chen C Jiang X METTL1-m^7^ G-EGFR/EFEMP1 axis promotes the bladder cancer development. Clin Translational Med (2021) 11(12):e675. 10.1002/ctm2.675 PMC8694502 34936728

[20] Huang M Long J Yao Z Zhao Y Zhao Y Liao J METTL1-Mediated m7G tRNA modification promotes lenvatinib resistance in hepatocellular carcinoma. Cancer Res (2023) 83(1):89–102. 10.1158/0008-5472.CAN-22-0963 36102722

[21] Liu Y Yang C Zhao Y Chi Q Wang Z Sun B. Overexpressed methyltransferase-like 1 (METTL1) increased chemosensitivity of colon cancer cells to cisplatin by regulating miR-149-3p/S100A4/p53 axis. Aging (Albany NY) (2019) 11(24):12328–44. 10.18632/aging.102575 31866582 PMC6949057

[22] Zhang Y Xu W Peng C Ren S Mustafe Hidig S Zhang C. Exploring the role of m7G modification in Cancer: mechanisms, regulatory proteins, and biomarker potential. Cell Signal (2024) 121:111288. 10.1016/j.cellsig.2024.111288 38971569

[23] Li L Yang Y Wang Z Xu C Huang J Li G. Prognostic role of METTL1 in glioma. Cancer Cell Int (2021) 21(1):633. 10.1186/s12935-021-02346-4 34838021 PMC8627054

[24] Peng W Fu J Zhou L Duan H. METTL1/FOXM1 promotes lung adenocarcinoma progression and gefitinib resistance by inhibiting PTPN13 expression. Cancer Med (2024) 13(13):e7420. 10.1002/cam4.7420 38967523 PMC11225164

[25] Wang S Han H Qian Y Ruan X Lin Z Li J Aberrant METTL1-mediated tRNA m^7^G modification alters B-cell responses in systemic autoimmunity in humans and mice. Nat Commun (2024) 15(1):10599. 10.1038/s41467-024-54941-4 39638793 PMC11621805

[26] Han H Yang C Ma J Zhang S Zheng S Ling R N^7^-methylguanosine tRNA modification promotes esophageal squamous cell carcinoma tumorigenesis via the RPTOR/ULK1/autophagy axis. Nat Commun (2022) 13(1):1478. 10.1038/s41467-022-29125-7 35304469 PMC8933395

[27] Long D Deng Z Zhao X Xu Y Li W Mo X m^7^G-modified mt-tRF3b-LeuTAA regulates mitophagy and metabolic reprogramming via SUMOylation of SIRT3 in chondrocytes. Biomaterials (2025) 314:122903. 10.1016/j.biomaterials.2024.122903 39454503

[28] Orellana EA Liu Q Yankova E Pirouz M De Braekeleer E Zhang W METTL1-mediated m^7^G modification of Arg-TCT tRNA drives oncogenic transformation. Mol Cell (2021) 81(16):3323–38.e14. 10.1016/j.molcel.2021.06.031 34352207 PMC8380730

[29] Dai Z Liu H Liao J Huang C Ren X Zhu W N^7^-Methylguanosine tRNA modification enhances oncogenic mRNA translation and promotes intrahepatic cholangiocarcinoma progression. Mol Cell (2021) 81(16):3339–55.e8. 10.1016/j.molcel.2021.07.003 34352206

[30] Zhao Z Qing Y Dong L Han L Wu D Li Y QKI shuttles internal m^7^G-modified transcripts into stress granules and modulates mRNA metabolism. Cell (2023) 186(15):3208–26.e27. 10.1016/j.cell.2023.05.047 37379838 PMC10527483

[31] Yu S Sun Z Ju T Liu Y Mei Z Wang C The m7G methyltransferase Mettl1 drives cardiac hypertrophy by regulating SRSF9-mediated splicing of NFATc4. Adv Sci (2024) 11(29):e2308769. 10.1002/advs.202308769 PMC11304317 38810124

[32] Liu C Dou X Zhao Y Zhang L Zhang L Dai Q IGF2BP3 promotes mRNA degradation through internal m^7^G modification. Nat Commun (2024) 15(1):7421. 10.1038/s41467-024-51634-w 39198433 PMC11358264

[33] Jin X Guan Z Hu N He C Yin P Gong Z Structural insight into how WDR4 promotes the tRNA N7-methylguanosine methyltransferase activity of METTL1. Cell Discov (2023) 9(1):65. 10.1038/s41421-023-00562-y 37369656 PMC10300002

[34] Ali RH Orellana EA Lee SH Chae YC Chen Y Clauwaert J A methyltransferase-independent role for METTL1 in tRNA aminoacylation and oncogenic transformation. Mol Cell (2025) 85(5):948–61.e11. 10.1016/j.molcel.2025.01.003 39892392 PMC11925124

[35] Zhang C Cui Y. N6-methyladenosine-induced METTL1 promotes tumor proliferation via CDK4. Biol Chem (2023) 405(3):217–28. 10.1515/hsz-2023-0260 37694982

[36] Sun Z Xu Y Si C Wu X Guo Y Chen C Targeting m7G-enriched circKDM1A prevents colorectal cancer progression. Mol Cancer (2024) 23(1):179. 10.1186/s12943-024-02090-z 39215345 PMC11363613

[37] Mi S Cai S Xue M Wu W. HIF-1α/METTL1/m^7^G axis is involved in CRC response to hypoxia. Biochem Biophysical Res Commun (2024) 693:149385. 10.1016/j.bbrc.2023.149385 38118310

[38] Chen J Yao S Sun Z Wang Y Yue J Cui Y The pattern of expression and prognostic value of key regulators for m^7^G RNA methylation in hepatocellular carcinoma. Front Genet (2022) 13:894325. 10.3389/fgene.2022.894325 36118897 PMC9478798

[39] Dong R Wang C Tang B Cheng Y Peng X Yang X WDR4 promotes HCC pathogenesis through N^7^-methylguanosine by regulating and interacting with METTL1. Cell Signal (2024) 118:111145. 10.1016/j.cellsig.2024.111145 38493882

[40] Zhao P Xia L Chen D Xu W Guo H Xu Y METTL1 mediated tRNA m^7^G modification promotes leukaemogenesis of AML via tRNA regulated translational control. Exp Hematol Oncol (2024) 13(1):8. 10.1186/s40164-024-00477-8 38268051 PMC10807064

[41] Lin H Liao F Liu J Yang Z Zhang J Cheng J Neuroblastoma susceptibility and association of N7-methylguanosine modification gene polymorphisms: multi-center case-control study. Pediatr Res (2025) 97(1):153–9. 10.1038/s41390-024-03318-w 38871802

[42] Liu J Deng C Lin H Zhang X Zhu J Zhou C Genetic variants of m7G modification genes influence neuroblastoma susceptibility. Heliyon (2024) 10(1):e23658. 10.1016/j.heliyon.2023.e23658 38173492 PMC10761801

[43] Yu D Yang J Wang B Li Z Wang K Li J New genetic insights into immunotherapy outcomes in gastric cancer via single-cell RNA sequencing and random forest model. Cancer Immunol Immunother (2024) 73(6):112. 10.1007/s00262-024-03684-8 38693422 PMC11063021

[44] Cui Y Hu Z Zhang C. RNA methyltransferase NSUN5 promotes esophageal cancer via 5-methylcytosine modification of METTL1. Mol Carcinogenesis (2024) 64(3):399–409. 10.1002/mc.23857 39601515

[45] Jiang H Liu Y Song H Xia J Tian Y Wang L METTL1 promotes colorectal cancer cell proliferation by attenuating CHEK2-induced G1/S phase arrest. Genes and Dis (2024) 11(2):579–81. 10.1016/j.gendis.2023.04.011 PMC10491903 37692515

[46] Zhang C Cui Y. Targeting TSPEAR-AS2 suppresses tumor growth and interferon signaling in esophageal cancer. Sci Rep (2024) 14(1):28768. 10.1038/s41598-024-80439-6 39567604 PMC11579022

[47] Chen Y Zhang X Li M Fu B Li H Yuan F METTL1-mediated m7G modification of NEK1 mRNA promotes the proliferation of oral squamous cell carcinoma. Biochim Biophys Acta (BBA) - Mol Basis Dis (2025) 1871(7):167961. 10.1016/j.bbadis.2025.167961 40562282

[48] Wang C He Y Fang X Zhang D Huang J Zhao S METTL1-modulated LSM14A facilitates proliferation and migration in glioblastoma via the stabilization of DDX5. iScience (2024) 27(7):110225. 10.1016/j.isci.2024.110225 39040050 PMC11261005

[49] Du D Zhou M Ju C Yin J Wang C Xu X METTL1-mediated tRNA m^7^G methylation and translational dysfunction restricts breast cancer tumorigenesis by fueling cell cycle blockade. J Exp Clin Cancer Res (2024) 43(1):154. 10.1186/s13046-024-03076-x 38822363 PMC11140866

[50] Ma X Qiu S Tang X Song Q Wang P Wang J TSPAN31 regulates the proliferation, migration, and apoptosis of gastric cancer cells through the METTL1/CCT2 pathway. Translational Oncol (2022) 20:101423. 10.1016/j.tranon.2022.101423 PMC9034387 35429902

[51] Ma J Zheng S An C Han H Li Q Huang Y Pathogenic mechanism and therapeutic intervention of impaired N^7^-methylguanosine (m^7^G) tRNA modification. Proc Natl Acad Sci U S A (2024) 121(45):e2405886121. 10.1073/pnas.2405886121 39471230 PMC11551429

[52] Zhang H He X Yang L Yang F Chen R Wen Z Trim45: an emerging E3 ubiquitin ligases in cancer. Cell Signal (2025) 134:111919. 10.1016/j.cellsig.2025.111919 40466841

[53] Han H Zheng S Lin S. N^7^-methylguanosine (m^7^G) tRNA modification: a novel autophagy modulator in cancer. Autophagy (2023) 19(1):360–2. 10.1080/15548627.2022.2077551 35574843 PMC9809925

[54] Wang C Wang W Han X Du L Li A Huang G. Methyltransferase-like 1 regulates lung adenocarcinoma A549 cell proliferation and autophagy via the AKT/mTORC1 signaling pathway. Oncol Lett (2021) 21(4):330. 10.3892/ol.2021.12591 33692862 PMC7933771

[55] Chen Z Zhu W Zhu S Sun K Liao J Liu H METTL1 promotes hepatocarcinogenesis via m^7^ G tRNA modification-dependent translation control. Clin Translational Med (2021) 11(12):e661. 10.1002/ctm2.661 PMC8666584 34898034

[56] Chen J Li K Chen J Wang X Ling R Cheng M Aberrant translation regulated by METTL1/WDR4-mediated tRNA N7-methylguanosine modification drives head and neck squamous cell carcinoma progression. Cancer Commun (2022) 42(3):223–44. 10.1002/cac2.12273 PMC8923133 35179319

[57] Wang Y Xiong G Cai W Tao Q. METTL1 facilitates ameloblastoma invasive growth via MAPK signaling pathway. Gene (2024) 905:148234. 10.1016/j.gene.2024.148234 38309318

[58] Li N Jing Y Xu L Wang M. METTL1 enhances RRP9 mRNA stability through m7G modification to drive colorectal tumorigenesis. Mol Carcinogenesis (2025) 64(5):858–69. 10.1002/mc.23892 39960239

[59] Zhang X Chen T Zhang F Shi H Li X Wang Z METTL1 coordinates cutaneous squamous cell carcinoma progression via the m7G modification of the ATF4 mRNA. Cell Death Discov (2025) 11(1):27. 10.1038/s41420-025-02304-3 39870616 PMC11772585

[60] Zhu S Wu Y Zhang X Peng S Xiao H Chen S Targeting N^7^-methylguanosine tRNA modification blocks hepatocellular carcinoma metastasis after insufficient radiofrequency ablation. Mol Ther (2023) 31(6):1596–614. 10.1016/j.ymthe.2022.08.004 35965412 PMC10278047

[61] Li T Chen Z Wang Z Lu J Chen D. Combined signature of N7-methylguanosine regulators with their related genes and the tumor microenvironment: a prognostic and therapeutic biomarker for breast cancer. Front Immunol (2023) 14:1260195. 10.3389/fimmu.2023.1260195 37868988 PMC10585266

[62] Liu Y Zhan Y Liu J Shen Z Hu Y Zhong L The 7-Methylguanosine (m7G) methylation METTL1 acts as a potential biomarker of clear cell renal cell carcinoma progression. Translational Oncol (2025) 51:102202. 10.1016/j.tranon.2024.102202 PMC11617297 39571491

[63] García-Vílchez R Añazco-Guenkova AM Dietmann S López J Morón-Calvente V D’Ambrosi S METTL1 promotes tumorigenesis through tRNA-derived fragment biogenesis in prostate cancer. Mol Cancer (2023) 22(1):119. 10.1186/s12943-023-01809-8 37516825 PMC10386714

[64] Yang J Bahcecioglu G Zorlutuna P. The extracellular matrix and vesicles modulate the breast tumor microenvironment. Bioengineering (Basel) (2020) 7(4):124. 10.3390/bioengineering7040124 33050609 PMC7712041

[65] Brassart-Pasco S Brézillon S Brassart B Ramont L Oudart JB Monboisse JC. Tumor microenvironment: extracellular matrix alterations influence tumor progression. Front Oncol (2020) 10:397. 10.3389/fonc.2020.00397 32351878 PMC7174611

[66] Golo M Newman PLH Kempe D Biro M. Mechanoimmunology in the solid tumor microenvironment. Biochem Soc Trans (2024) 52(3):1489–502. 10.1042/BST20231427 38856041

[67] Garcia-Vilchez R Anazco-Guenkova AM Lopez J Dietmann S Tome M Jimeno S N7-methylguanosine methylation of tRNAs regulates survival to stress in cancer. Oncogene (2023) 42(43):3169–81. 10.1038/s41388-023-02825-0 37660182 PMC10589097

[68] Zeng X Liao G Li S Liu H Zhao X Li S Eliminating METTL1-mediated accumulation of PMN-MDSCs prevents hepatocellular carcinoma recurrence after radiofrequency ablation. Hepatology (2023) 77(4):1122–38. 10.1002/hep.32585 35598182

[69] Xu J You Z Zhu Z Liu M Zhang Z Xu P Integrative analysis of m7G methylation-associated genes prognostic signature with immunotherapy and identification of LARP1 as a key oncogene in head and neck squamous cell carcinoma. Front Immunol (2025) 16:1520070. 10.3389/fimmu.2025.1520070 40018039 PMC11864954

[70] Wang F Yang C Zheng F Yan Y Li G Feng Y METTL1 mediates PKM m7G modification to regulate CD155 expression and promote immune evasion in colorectal cancer. J Transl Med (2024) 22(1):1161. 10.1186/s12967-024-05991-1 39741310 PMC11686999

[71] Liu H Zeng X Ren X Zhang Y Huang M Tan L Targeting tumour-intrinsic N^7^-methylguanosine tRNA modification inhibits MDSC recruitment and improves anti-PD-1 efficacy. Gut (2023) 72(8):1555–67. 10.1136/gutjnl-2022-327230 36283801

[72] Xu F Cai D Liu S He K Chen J Qu L N7-methylguanosine regulatory genes well represented by METTL1 define vastly different prognostic, immune and therapy landscapes in adrenocortical carcinoma. Am J Cancer Res (2023) 13(2):538–68. 36895966 PMC9989616

[73] Xu J Cen X Yao Y Zhao S Li W Zhang W Identification of six N7-methylguanosine-related miRNA signatures to predict the overall survival and immune landscape of triple-negative breast cancer through *in silico* analysis. J Oncol (2022) 2022:2735251. 10.1155/2022/2735251 36199792 PMC9529398

[74] Liu Y Zhu E Lei Y Luo A Yan Y Cai M Diagnostic values of METTL1-related genes and immune characteristics in systemic lupus erythematosus. J Inflamm Res (2023) 16:5367–83. 10.2147/JIR.S431628 38026241 PMC10661937

[75] Denton AE Roberts EW Fearon DT. Stromal cells in the tumor microenvironment. Adv Exp Med Biol (2018) 1060:99–114. 10.1007/978-3-319-78127-3_6 30155624

[76] Cao H Gao S Jogani R Sugimura R. The tumor microenvironment reprograms immune cells. Cell Reprogramming (2022) 24(6):343–52. 10.1089/cell.2022.0047 36301256

[77] Zhao Y Shen M Wu L Yang H Yao Y Yang Q Stromal cells in the tumor microenvironment: accomplices of tumor progression? Cell Death Dis (2023) 14(9):587. 10.1038/s41419-023-06110-6 37666813 PMC10477351

[78] Weng X Huang Y Fu Z Liu X Xie F Wang J METTL1-driven nucleotide metabolism reprograms the immune microenvironment in hepatocellular carcinoma: a multi-omics approach for prognostic biomarker discovery. Front Immunol (2025) 16:1582203. 10.3389/fimmu.2025.1582203 40330476 PMC12052905

[79] Pandolfini L Barbieri I Bannister AJ Hendrick A Andrews B Webster N METTL1 promotes let-7 MicroRNA processing via m7G methylation. Mol Cell (2019) 74(6):1278–90.e9. 10.1016/j.molcel.2019.03.040 31031083 PMC6591002

[80] Guo Z Li Z Guo J Gan L Mo H Zhang J A N7-methylguanosine modified circular RNA, circIPP2A2, promotes malignant behaviors in hepatocellular carcinoma by serving as a scaffold in modulating the Hornerin/PI3K/AKT/GSK3β axis. Cell Death Dis (2024) 15(11):868. 10.1038/s41419-024-07248-7 39616161 PMC11608253

[81] Nai F Flores Espinoza MP Invernizzi A Vargas-Rosales PA Bobileva O Herok M Small-molecule inhibitors of the m7G-RNA writer METTL1. ACS Bio Med Chem Au (2024) 4(2):100–10. 10.1021/acsbiomedchemau.3c00030 PMC11027120 38645929

[82] Zhao R Chen J Wang Y Xiao H Mei P Lin W Prognostic roles of dysregulated METTL3 protein expression in cancers and potential anticancer value by inhibiting METTL3 function. Fundam and Clin Pharmacol (2024) 38(5):924–39. 10.1111/fcp.13020 38849971

[83] Wu Z Smith AR Qian Z Zheng G. Patent landscape of small molecule inhibitors of METTL3 (2020-present). Expert Opin Ther Patents (2024) 35:305–20. 10.1080/13543776.2024.2447056 PMC12198425 39721070

[84] Xu R Du A Deng X Du W Zhang K Li J tsRNA-GlyGCC promotes colorectal cancer progression and 5-FU resistance by regulating SPIB. J Exp Clin Cancer Res (2024) 43(1):230. 10.1186/s13046-024-03132-6 39153969 PMC11330149

[85] Tong F Wang Y Gao H. Progress and challenges in the translation of cancer nanomedicines. Curr Opin Biotechnol (2024) 85:103045. 10.1016/j.copbio.2023.103045 38096768

[86] Zhao Y Gong J Liu H Huang H Tan WS Cai H. A chemically defined, mechanically tunable, and bioactive hyaluronic acid/alginate double-network hydrogel for liver cancer organoid construction. Int J Biol Macromolecules (2024) 282(Pt 2):136707. 10.1016/j.ijbiomac.2024.136707 39442832

[87] Lee SH Kim K Lee E Lee K Ahn KH Park H Prediction of TKI response in EGFR-mutant lung cancer patients-derived organoids using malignant pleural effusion. npj Precision Oncol (2024) 8(1):111. 10.1038/s41698-024-00609-7 PMC11109121 38773241

[88] Verghese M Wilkinson E He YY. Recent advances in RNA m^6^A modification in solid tumors and tumor immunity. Cancer Treat Res (2023) 190:95–142. 10.1007/978-3-031-45654-1_4 38113000

[89] Yang J Xu J Wang W Zhang B Yu X Shi S. Epigenetic regulation in the tumor microenvironment: molecular mechanisms and therapeutic targets. Signal Transduction Targeted Ther (2023) 8(1):210. 10.1038/s41392-023-01480-x PMC10203321 37217462

[90] Wu C Li L Tang Q Liao Q Chen P Guo C Role of m^6^A modifications in immune evasion and immunotherapy. Med Oncol (2024) 41(6):159. 10.1007/s12032-024-02402-9 38761335

[91] Dai P Chen Y Zhang X Liu L Cheng Z. MRPL13 is a metastatic and prognostic marker of breast cancer: a silico analysis accompanied with experimental validation. Gene (2025) 932:148908. 10.1016/j.gene.2024.148908 39218414

